# A miRNA-disease association prediction model based on tree-path global feature extraction and fully connected artificial neural network with multi-head self-attention mechanism

**DOI:** 10.1186/s12885-024-12420-5

**Published:** 2024-06-05

**Authors:** Hou Biyu, Li Mengshan, Hou Yuxin, Zeng Ming, Wang Nan, Guan Lixin

**Affiliations:** 1 College of Physics and Electronic Information, Gannan Normal University, Ganzhou, Jiangxi 341000 China; 2 College of Computer Science and Engineering, Shanxi Datong University, Datong, Shanxi 037000 China; 3 College of Life Sciences, Jiaying University, Meizhou, Guangdong 514000 China

**Keywords:** Association tree, Multi-head self-attention mechanism, miRNA-disease association, Deep learning, Cancer

## Abstract

**Background:**

MicroRNAs (miRNAs) emerge in various organisms, ranging from viruses to humans, and play crucial regulatory roles within cells, participating in a variety of biological processes. In numerous prediction methods for miRNA-disease associations, the issue of over-dependence on both similarity measurement data and the association matrix still hasn’t been improved. In this paper, a miRNA-Disease association prediction model (called TP-MDA) based on tree path global feature extraction and fully connected artificial neural network (FANN) with multi-head self-attention mechanism is proposed. The TP-MDA model utilizes an association tree structure to represent the data relationships, multi-head self-attention mechanism for extracting feature vectors, and fully connected artificial neural network with 5-fold cross-validation for model training.

**Results:**

The experimental results indicate that the TP-MDA model outperforms the other comparative models, AUC is 0.9714. In the case studies of miRNAs associated with colorectal cancer and lung cancer, among the top 15 miRNAs predicted by the model, 12 in colorectal cancer and 15 in lung cancer were validated respectively, the accuracy is as high as 0.9227.

**Conclusions:**

The model proposed in this paper can accurately predict the miRNA-disease association, and can serve as a valuable reference for data mining and association prediction in the fields of life sciences, biology, and disease genetics, among others.

**Graphical Abstract:**

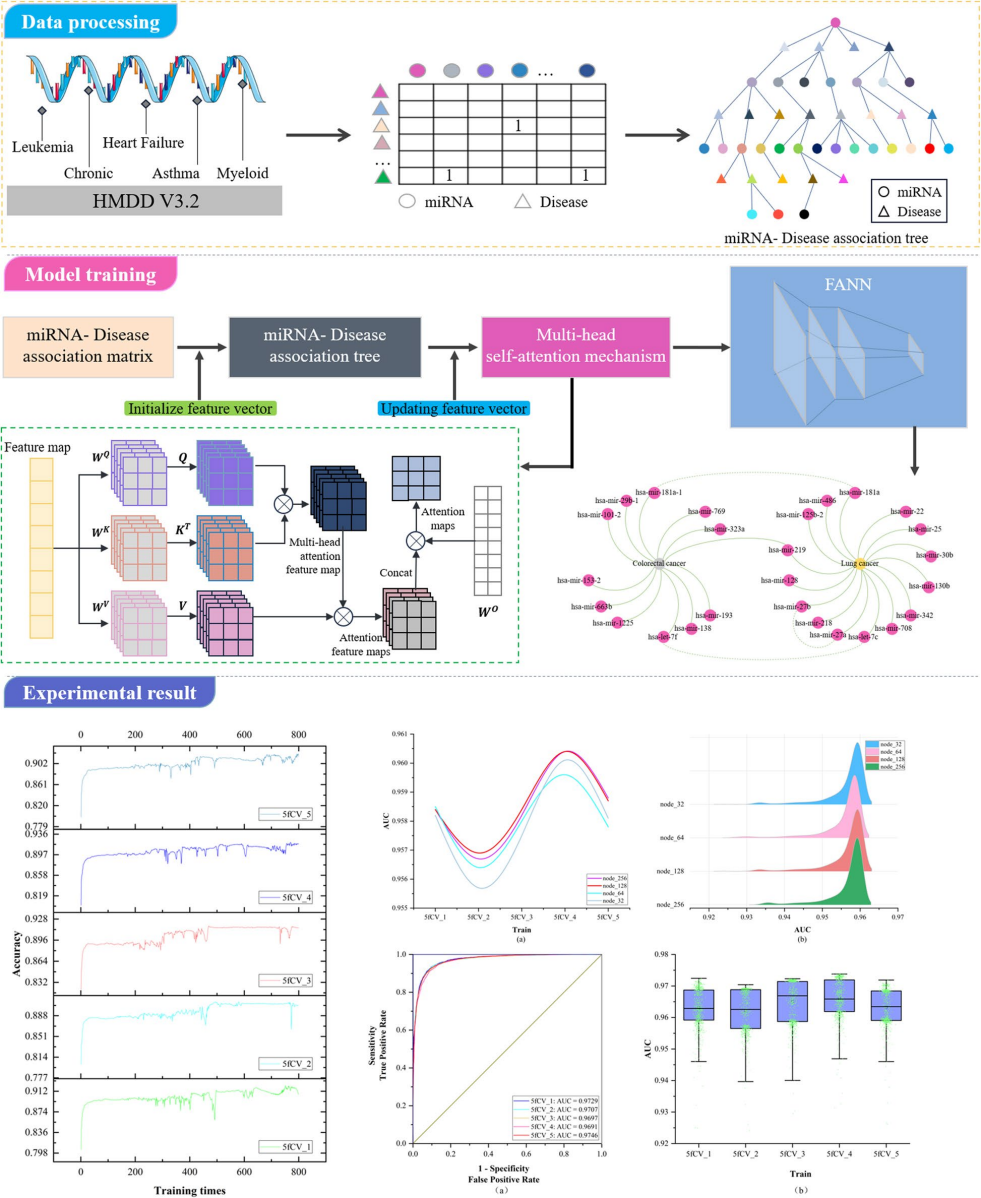

## Introduction

MicroRNA (miRNA) is a class of short 20–24 nucleotide non-coding RNA molecules that play critical regulatory roles in cells [1, 2]. They form a complex regulatory network and are involved in various biological processes such as cell proliferation, differentiation and apoptosis [3]. In addition, miRNA is closely related to the occurrence and development of cancer, cardiovascular diseases, nervous system diseases and other diseases [4–7]. For example, cancer stem cell-like cells (CSCs) are increasingly recognized as key cell tumor populations that drive not only tumorigenesis, but also cancer progression, treatment resistance, and metastatic recurrence. Existing evidence suggests that different metabolic pathways regulated by let-7 miRNA can impact CSC self-renewal, differentiation, and treatment resistance [8]. Therefore, in-depth research on the association between miRNAs and diseases is of great importance for understanding cellular regulatory mechanisms, discovering new therapeutic targets, and developing relevant biomedical applications [9–12].

With the continuous advancement of bioinformatics and the advent of the artificial intelligence era, researchers are increasingly using machine learning and deep learning algorithms to predict miRNA-disease associations [13–15]. It can provide validation guidance for biological experiments, thereby conserving resources and further advancing the field of miRNA and disease association prediction [16–18].It also has the potential to drive further advances in miRNA-disease association prediction. Based on different prediction strategies, existing methods can be categorized into four types: machine learning-based methods, information propagation-based methods, scoring function-based methods, and matrix transformation-based methods [19, 20]. Machine learning-based prediction methods have recently become a focus and are gaining popularity among researchers [21, 22]. Yu et al. [23] constructed a heterogeneous information network including miRNA, diseases, and genes. They defined seven symmetric meta-paths based on different semantic interpretations. After initializing the feature vectors for all nodes, they extracted and aggregated the vector information carried by all nodes on meta-path instances and updated the starting node’s feature vector. Then, they aggregated the vector information obtained from nodes on different meta-paths. Finally, they used miRNA and disease embedding feature vectors to compute their association scores. Xie et al. [24] constructed miRNA-disease bias scores using aggregated hierarchical clustering. A bipartite network recommendation algorithm was then used to assign transfer weights based on these bias ratings to predict potential miRNA-disease associations. Chen et al. [25] combined known miRNA and disease similarities to establish transfer weights and appropriately configured initial information. They then used a two-stage bipartite network algorithm to infer potential miRNA-disease associations.

In the study of miRNA-disease associations, there are two areas that need improvement: (1) The ability to capture indirect association features is inadequate. Among various computational methods, researchers use miRNA-disease heterogeneous networks to structure miRNA-disease association data and then extract feature vectors from the heterogeneous network. However, the associations within the heterogeneous network are limited to direct relationships between miRNAs and diseases, and their ability to capture indirect associations is often weak. This limitation may result in reduced model performance. (2) Over-reliance on similarity measurement data. Many computational methods rely on similarity information such as miRNA similarity and disease similarity for model training. The reliance on similarity data can, to a certain extent, influence the discriminative ability of the model and have an impact on its predictive accuracy.

To address the first issue, this paper investigates a data organization approach based on a tree-like topological structure. It represents miRNAs or diseases as root nodes and then searches for all related diseases or miRNAs as the second layer of the tree. All miRNAs or disease nodes associated with each disease or miRNA in the second layer are then found in the dataset. This process is repeated until the entire dataset has been thoroughly searched. At this point, there is a unique tree with the miRNA or disease as the root node, called the miRNA-disease association tree. This tree contains all association relationships related to that miRNA or disease within the dataset. Next, the vector information carried by all nodes on each path instance is extracted on the paths of the tree. Vector information obtained from nodes on different tree-paths is aggregated to generate feature vectors for model training. The miRNA-disease association tree has the potential to improve the capture of indirect association features. In response to Problem 2, since the similarity of data is often subjective based on some human-set metric, these data may produce misleading results in some cases, which in turn affects the performance of the algorithm. In contrast to similarity measures, multi-head self-attention mechanisms better capture long-distance dependencies in input sequences by allowing the model to focus on information from different locations, which in turn improves the predictive performance of the model. In this paper, we explore the use of the multi-head self-attention mechanism to fully extract the long dependencies carried by association trees, avoiding the bias created by using similarity measures and overcoming the problem of over-reliance on similarity measure data. As a result, the paper introduces a miRNA-disease association prediction model. This model uses a multi-head self-attention mechanism for comprehensive feature extraction on the tree-paths. It then trains the dataset using the Fully Connected Artificial Neural Network (FANN) model in a 5-fold cross-validation experiment. This model is referred to as TP-MDA.

## Materials and methods

### Establishing the Association Matrix

Based on the miRNA-disease association information, remove duplicate, missing, and invalid data in order to construct the miRNA-disease association matrix. Given m miRNAs, M={m_1_、…、m_i_、…、m_m_ },and n diseases, D = {d_1_, …, d_j_, …, d_n_},the miRNA-disease association matrix is defined as R, where R∈R_m×n_, as shown in Eq. (1):

1 $$R_{ij}\;=\left\{\begin{array}{l}1,\;miRNA\;have\;been\;linked\;to\;disease\;\\0,\;The\;relationship\;between\;miRNA\;and\;disease\;is\;unknown\end{array}\right.$$

**Fig. 1:**
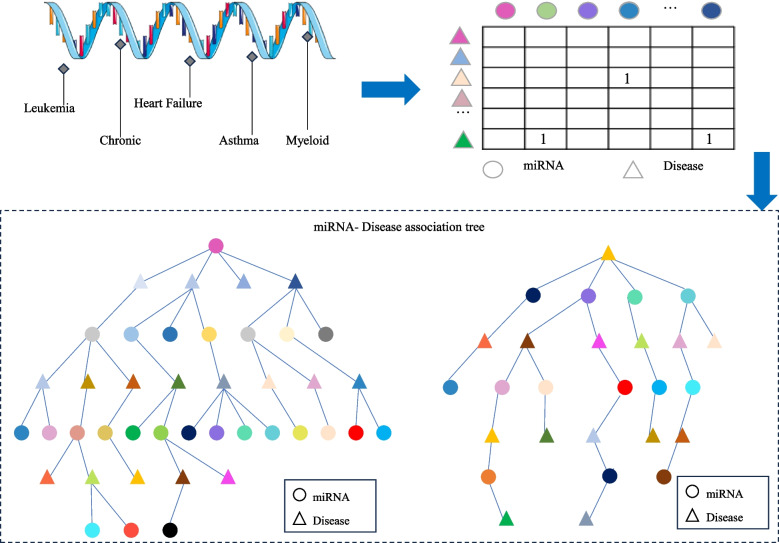
The construction of the association tree

Subsequently, the miRNA-disease association tree is constructed by continuously exploring the association matrix. The process of association tree construction is shown in Fig. 1.

### Multi-head self-attention mechanism

The self-attention mechanism is a special type of attention mechanism used to handle relationships between different positions in sequence data. The multi-head self-attention mechanism is a common extension of the attention mechanism in deep learning that employs multiple attention heads at the same level, allowing for the fusion of different attention weights. In this paper, a multi-head self-attention mechanism is used to process the feature vectors extracted from the miRNA-disease association tree. The self-attention mechanism is as shown in Eqs. (2) and (3):

2 $$\left\{\begin{array}{l}Q\;=\;X\ast W^Q\\K\;=\;X\ast W^K\\V\;=\;X\ast W^V\end{array}\right.$$

3 $$\mathrm{Attention}(\mathrm Q,\mathrm K,\mathrm V)=\mathrm{softmax}\left(\frac{Q\mathit\ast K^{\mathit T}}{\sqrt{d_k}}\right)\mathrm V$$

In the equations, X represents the vector information extracted from the miRNA-disease association tree, and Q, K, V represent the query matrix, key matrix, and value matrix, respectively. These three matrices are obtained by linear transformations of X using W^Q^, W^K^, and W^V^. Here, d_k_ represents the dimension of the query, key, or value.

The multi-head self-attention mechanism transforms the linear matrices from a set ($${\varvec{W}}^{\varvec{Q}},$$$${\varvec{W}}^{\varvec{K}},$$$${\varvec{W}}^{\varvec{V}}$$) to multiple sets {($${\varvec{W}}_{0}^{\varvec{Q}}$$, $${\varvec{W}}_{0}^{\varvec{K}}$$, $${\varvec{W}}_{0}^{\varvec{V}}$$), …, ( $${\varvec{W}}_{\varvec{i}}^{\varvec{Q}}$$, $${\varvec{W}}_{\varvec{i}}^{\varvec{K}}$$, $${\varvec{W}}_{\varvec{i}}^{\varvec{V}}$$) }. Different sets of linear matrices with random initialization ($${\varvec{W}}^{\varvec{Q}}$$, $${\varvec{W}}^{\varvec{K}}$$, $${\varvec{W}}^{\varvec{V}}$$) can map the input vectors to different subspaces, allowing the model to understand input information from different spatial dimensions. The multi-head attention mechanism is represented as shown in Eqs. (4) and (5):

4 $$head_i=\;Attention\left(QW_i^Q\mathit,\mathit\;KW_{\mathit i}^{\mathit K}\mathit,\mathit\;VW_i^V\right)$$

5 $$MultiHead\left(\mathrm Q,\mathrm K,\mathrm V\right)=Concat\left(head_{1,\;\cdots\;,\;\;}head_h\right)\;\ast\;W^O$$

In these equations, ${\varvec{W}}_{\varvec{i}}^{\varvec{Q}}$, ${\varvec{W}}_{\varvec{i}}^{\varvec{K}}$, ${\varvec{W}}_{\varvec{i}}^{\varvec{V}}$ represent the query matrix, key matrix, and value matrix for the i-th head, where h is the number of heads. ${\varvec{W}}^{\varvec{O}}$ is the linear transformation matrix used to map the output of the multi-head self-attention mechanism into the same dimensional space.

**Fig. 2:**
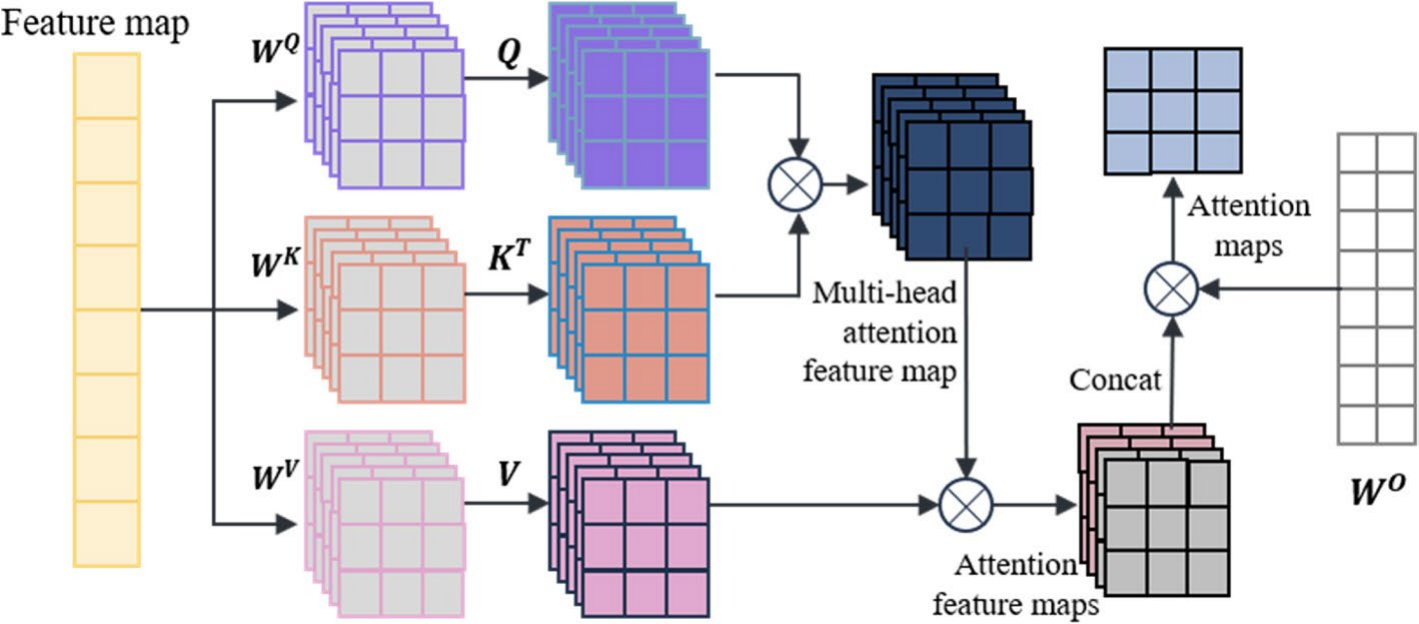
Multi-head self-attention mechanism

The key point of the self-attention mechanism is the ability to consider information about all other elements in the sequence while calculating the association between each element, rather than considering only a fixed number of adjacent elements as in traditional fixed window or convolution operations. Therefore, the self-attention mechanism can effectively manage long dependencies, allowing for improved capture of semantic information within the sequence, and there are numerous long dependencies to be addressed within the miRNA-disease association tree. In this paper, after the initial feature vector information is extracted from the tree nodes, the multi-head self-attention mechanism is used for information processing, resulting in the acquisition of the updated feature vector, which is used as input for model training. The operation principle is shown in Fig. 2.

### TP-MDA model

**Fig. 3:**
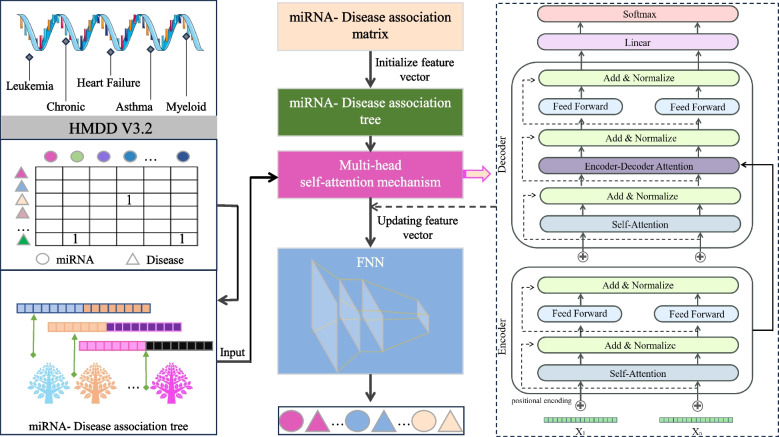
Model diagram

In the TP-MDA model, the miRNA-disease association matrix is transformed into a miRNA-disease association tree to explore long dependencies between nodes. A multi-head self-attention mechanism network is used to aggregate and extract information along the tree-paths. The outputs are concatenated to create feature vectors, which are subsequently used as input for training the FANN model. The schematic diagram of the TP-MDA model is illustrated in Fig. 3.

In this paper, a Fully Connected Artificial Neural Network (FANN) is used to train the data. In addition to the input and output layers, three hidden layers have been configured. The ReLU (Rectified Linear Unit) function is used as the activation function, as depicted in Eq. (6):

6 $$\mathrm{ReLU}\;=\;\max\left(0,\;x\right)$$

For the output layer, a sigmoid function is set as the activation function, as shown in Eq. (7):

7 $$\mathrm{sigmoid}\;\left(\mathrm X\right)\;=\frac1{1+\exp\;\left(-\mathrm x\right)}$$

The loss function used is cross-entropy loss, and the TP-MDA model is trained using the Adam optimizer. The learning rate is set to 0.000001 and the number of iterations is set to 800. The prediction results of the model represent the predicted values for miRNA-disease associations.

### Data source and model evaluation

The data in this paper is sourced from the Human microRNA Disease Database (HMDD, v4.0, http://www.cuilab.cn/hmdd). The database is a widely used miRNA-disease association database that not only compiles experimentally validated miRNA-disease associations, but also enables normalized naming of miRNAs. The original dataset obtained from this database download contains 35,547 miRNA-disease association information. Since this data is a large dataset consisting of five assay methods, there are a certain number of duplicate entries. After removing duplicate entries and irrelevant information, the miRNA-disease association information is obtained, as shown in Table 1.

**Table 1:** Experimental data of TP-MDA model

DataSet	miRNA	Disease	Correlation	Correlation rate
HMDD v3.2	1207	889	21,152	1.971%

As shown in Table 1, a total of 21,152 miRNA-disease associations were obtained after preprocessing the dataset. A large sparse matrix with a dimension of 1207*889 was obtained from the construction of these data, and the miRNA-disease association tree was subsequently constructed by traversal operations on the matrix. During the training process of the TP-MDA model, samples with the same number of positive samples were randomly selected as negative samples among all unknown samples. In order to increase the generalization ability of the TP-MDA model to different sets of negative samples, it is set in the subsequent 5-fold cross-validation experiments that the negative samples selected in each experiment are not duplicated with the previous fold experiment.

During model training, a 5-fold cross-validation is used for training and validation, as shown in Eq. (8):

8 $$CV_{\left(k\right)}\;=\frac1k{\textstyle\sum_{i=1\;\;}^k}MSE_i$$

In the equation, k = 5 indicates the use of 5-fold cross-validation in the experiment, and MSE represents Mean Squared Error, a common measure used to evaluate the model’s performance.

When plotting the Receiver Operating Characteristic (ROC) curve, the data includes one-fifth of the positive samples and an equal number of randomly selected negative samples for validation. The true positive rate (TPR) and false positive rate (FPR) are calculated using the prediction results from this data, as shown in Eqs. (9) and (10):

9 $$TPR\;=\frac{\mathrm{TP}}{TP+FN\;\;\;}$$

In the equations, TP represents the number of correctly identified positive samples, while FN represents the number of incorrectly identified positive samples.

10 $$FPR\;=\frac{\mathrm{FP}}{FP+TN\;\;\;}$$

Where FP represents the number of incorrectly identified negative samples, and TN represents the number of correctly identified negative samples. By setting different classification thresholds, FPR and TPR are represented on the horizontal and vertical axes to create the Receiver Operating Characteristic (ROC) curve, which serves as one of the performance evaluation metrics for the model. The area under the ROC curve, defined as AUC, is typically considered an indicator of classifier performance, with larger AUC values associated with better classifier performance.

Additionally, accuracy is employed as one of the model evaluation metrics. In this paper, accuracy is calculated using validation data, as illustrated in Eq. (11):

11 $$ACC={\textstyle\frac{TP}{TP+FN}}$$

The TP-MDA model consists of the following three parts: (1) Data processing: The miRNA-disease association data are transformed into an association matrix. The miRNA-disease association tree is constructed by continuously searching through the association matrix. In this paper, a miRNA-disease association tree is defined, with separate trees constructed using miRNA and disease as root nodes. All diseases or miRNAs associated with them in the association matrix are considered as the next-layer child nodes. Each disease or miRNA node is then traversed to identify its associated miRNAs or diseases. This process is repeated until the entire dataset has been completely traversed, yielding a distinct association tree with the miRNA or disease as the root node. (2) Feature Extraction: In the association tree, there are many long dependencies. The multi-head attention mechanism is employed to extract information held by the nodes of the tree structure. The information from different types of root nodes in the association tree is extracted separately and then concatenated to form feature vectors for potential miRNA-disease association prediction models. (3) Model training: The feature vectors are fed into a five-layer fully connected neural network whose output represents the miRNA-disease association score.

## Results

### Analysis of node number optimization experiment results

**Fig. 4:**
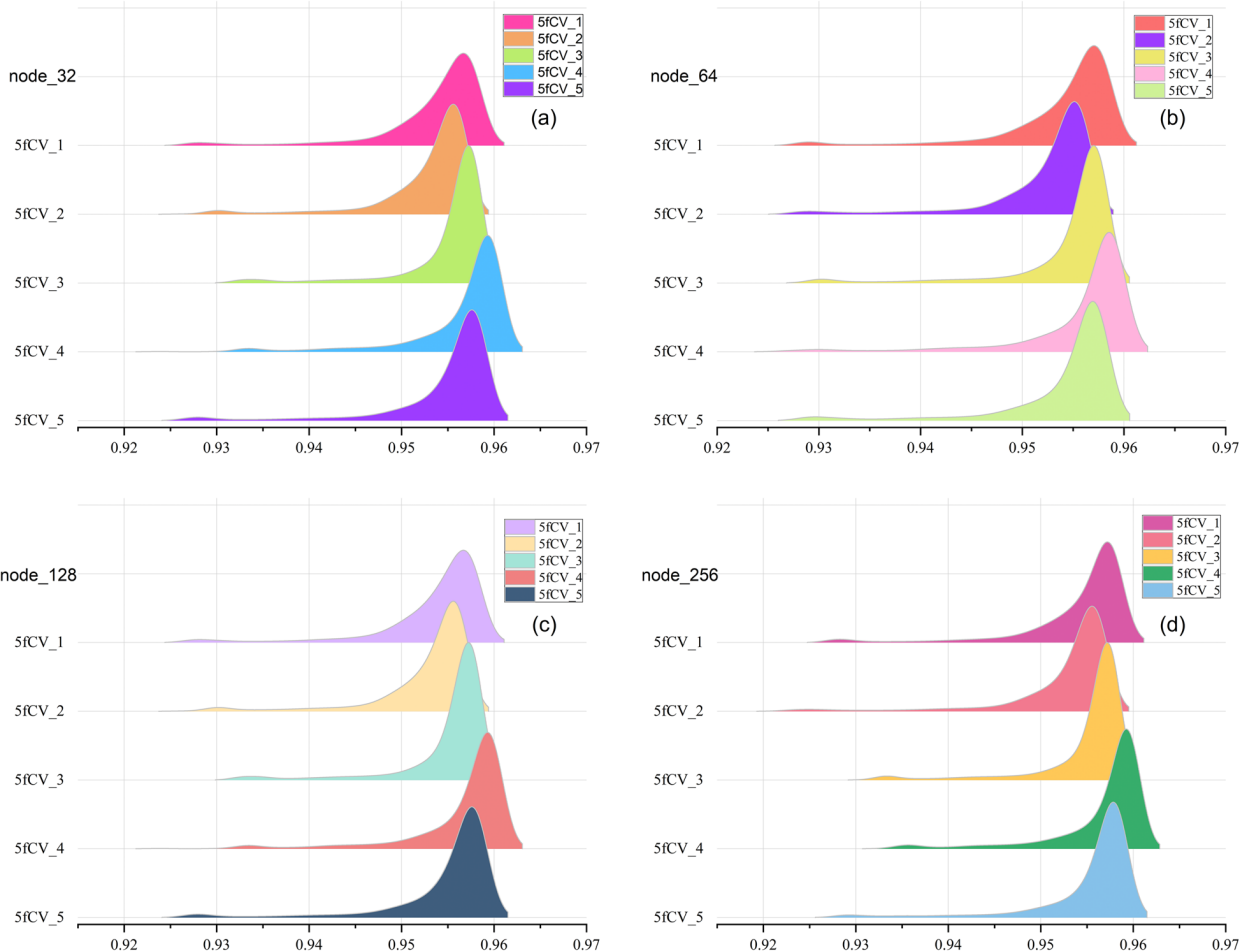
AUC statistical results of 800 rounds of experiments with different number of nodes. **a** AUC statistics when the number of nodes is 32, **b** AUC statistics when the number of nodes is 64, **c** AUC statistics when the number of nodes is 128, **d** AUC statistics when the number of nodes is 256

In this paper, the data is trained using a 5-layer fully connected neural network, and the number of neurons in each fully connected layer is a critical parameter, especially in the last fully connected layer. The number of neurons in the final fully connected layer determines the dimension of the potential miRNA-disease interaction vectors, and this is a critical factor in predicting miRNA-disease associations [26]. However, running experiments with different hyperparameter combinations using LOOCV can be time-consuming. To save experimental resources, we only compare the performance of different numbers of neurons in the last fully connected layer. Therefore, we select different numbers of nodes for optimization with the goal of obtaining better parameters for model training. The AUC values of the model under different numbers of nodes are shown in Fig. 4.

In the ridge plot, each peak represents one fold of the experiment, and it summarizes the AUC values during the 800 training rounds. The higher the peak, the more training rounds the model has reached at that specific AUC value, and peaks located to the right indicate a larger median in the statistical data, which corresponds to better model performance. As the number of nodes increases, the statistical results of the AUC value under the 5-fold cross-validation experiment are basically the same. The experimental results show that the best performance is observed in the fourth fold, while the second fold shows the worst performance. The median of all peaks is above 0.95, and in the fourth replicate there are more AUC values reaching 0.96. The results indicate that the HMDD v3.2 dataset can be effectively used for stable predictions in the TP-MDA model, which shows promising predictive performance in miRNA-disease association experiments. This suggests that the TP-MDA algorithm has superior performance in predicting miRNA-disease associations.

**Fig. 5:**
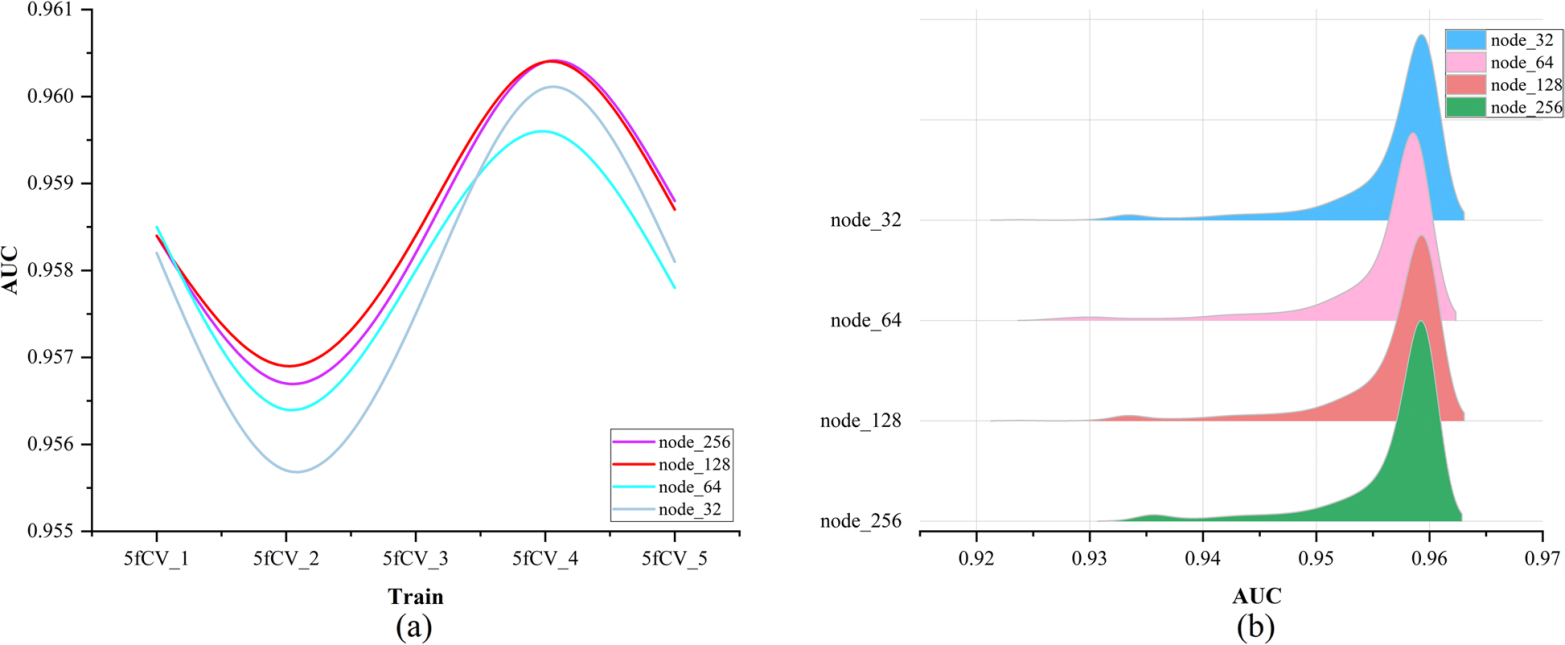
The final experimental results under different nodes. **a** The final AUC experimental results under different number of nodes, **b** The final AUC statistics under different node numbers

The experimental results for different numbers of nodes are statistically analyzed. A more detailed examination of all the results from the fourth fold in Fig. 4 is performed to determine the optimal number of nodes. The statistical results are shown in Fig. 5.

The trend of AUC values remains consistent as the number of nodes changes in Fig. 5a. When the number of nodes is set to 128, the AUC performance is superior to that at other node counts and is optimal in the second, third, and fourth fold experiments. The models with 32 and 128 nodes perform similarly in Fig. 5b. By analyzing Fig. 5a and b together, it can be concluded that the model performs better when the number of nodes is 128.

### Analysis of learning rate optimization experiment results

**Fig. 6:**
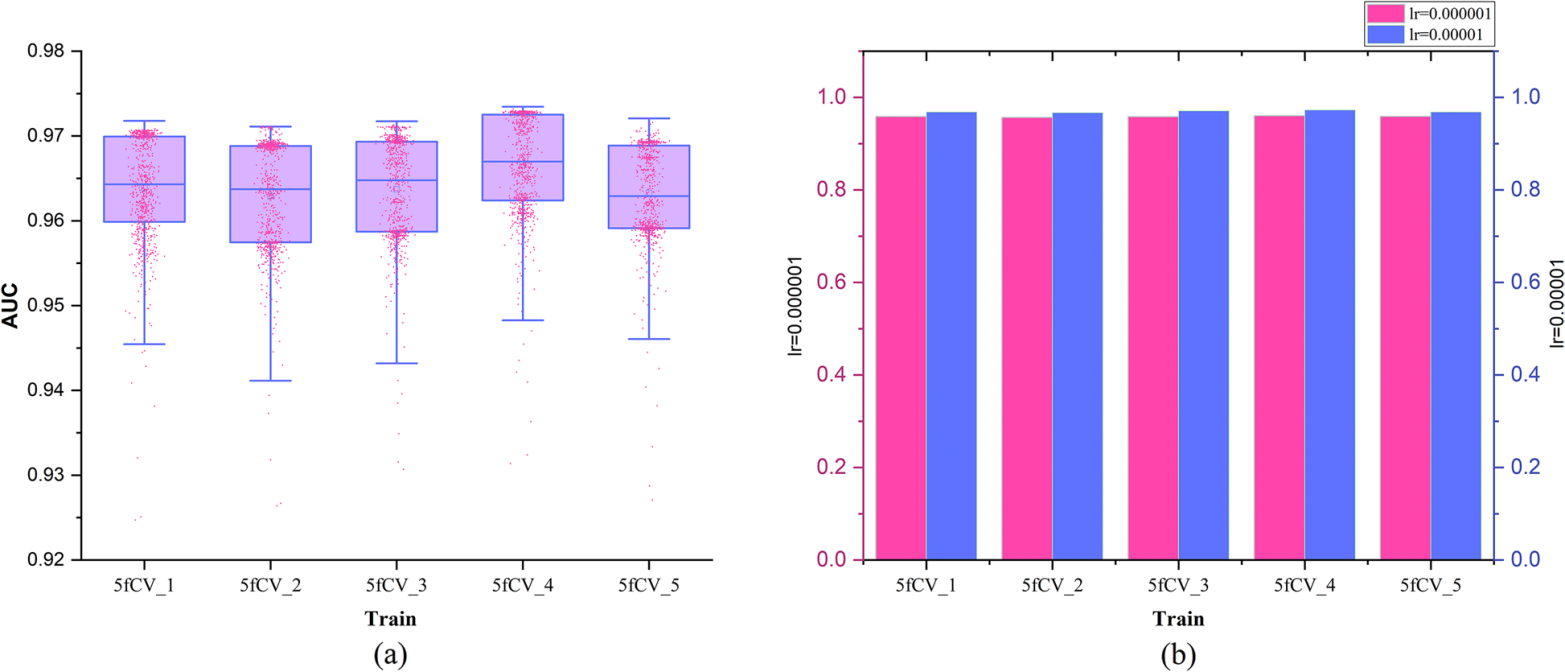
Experimental results at different learning rates. **a** When the learning rate is equal to 0.00001, the AUC statistical result of 800 rounds of experiments, **b** The final experimental results of AUC under different learning rates

The learning rate is crucial for determining whether the network model can converge to the optimal point, so a learning rate optimization process is carried out. The results are shown in Fig. 6.

During the learning rate optimization process, other parameters were held constant while the learning rate was changed. When the learning rate was set to 0.000001, it produced the same results as the model experiments shown in Fig. 4d. In the experimental results shown in Fig. 4d, there were no model AUC values that exceeded 0.97. In the experimental results shown in Fig. 6a, some of the AUC values exceeded 0.97, at which point the learning rate (lr) was set to 0.00001. This indicates that when the learning rate is set to 0.00001, the model’s predictive performance improved over multiple rounds of experiments. Figure 6b compares the final AUC values of the model under different learning rates, and the results show that the AUC values are consistently higher when lr = 0.00001 in the 5-fold cross-validation experiments compared to when lr = 0.000001. By optimizing the learning rate under the same experimental conditions, it was found that the prediction performance of the model is better when lr = 0.00001. The learning rate is crucial for TP-MDA to find the optimal point, and a more suitable learning rate parameter can improve the accuracy of miRNA-disease association prediction.

### Comparison between association tree and association matrix in experiments

**Fig. 7:**
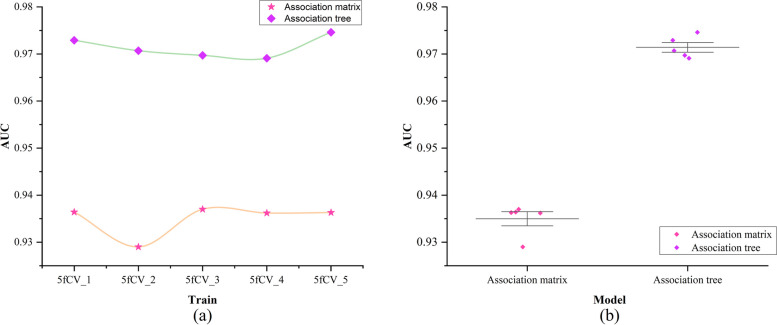
Comparison of AUC values of association matrix and association tree. **a** Comparison of AUC results under 5-fold cross-validation experiment using association matrix and association tree as input, **b** Statistics of AUC results under 5-fold cross-validation experiment using association matrix and association tree as input

To validate whether the improvement of the miRNA-disease association tree has a positive impact on the model, this paper conducted experiments with the same experimental parameters on the miRNA-disease association matrix. In these experiments, the rows and columns of the association matrix were concatenated to form a vector. Attention mechanisms were then used to extract feature vectors, and the resulting vectors were fed into a fully connected neural network for training. A comparison of the model training results using the miRNA-disease association matrix and the miRNA-disease association tree as inputs is shown in Fig. 7.

The green line represents the AUC results obtained using the miRNA-disease association tree as input, while the yellow line represents the AUC results obtained using the miRNA-disease association matrix as input, as shown in Fig. 7a. In the experiments with 5-fold cross-validation using the association tree as input, the AUC values exceeded 0.97, while using the association matrix as input did not reach 0.94. The model using the miRNA-disease association tree shows significantly better and more stable performance under 5-fold cross-validation, as shown in Fig. 7b. The experimental results show a significant improvement in predictive performance when using the association tree as input, indicating the superiority of the TP-MDA model in predicting potential miRNA-disease associations.

**Fig. 8:**
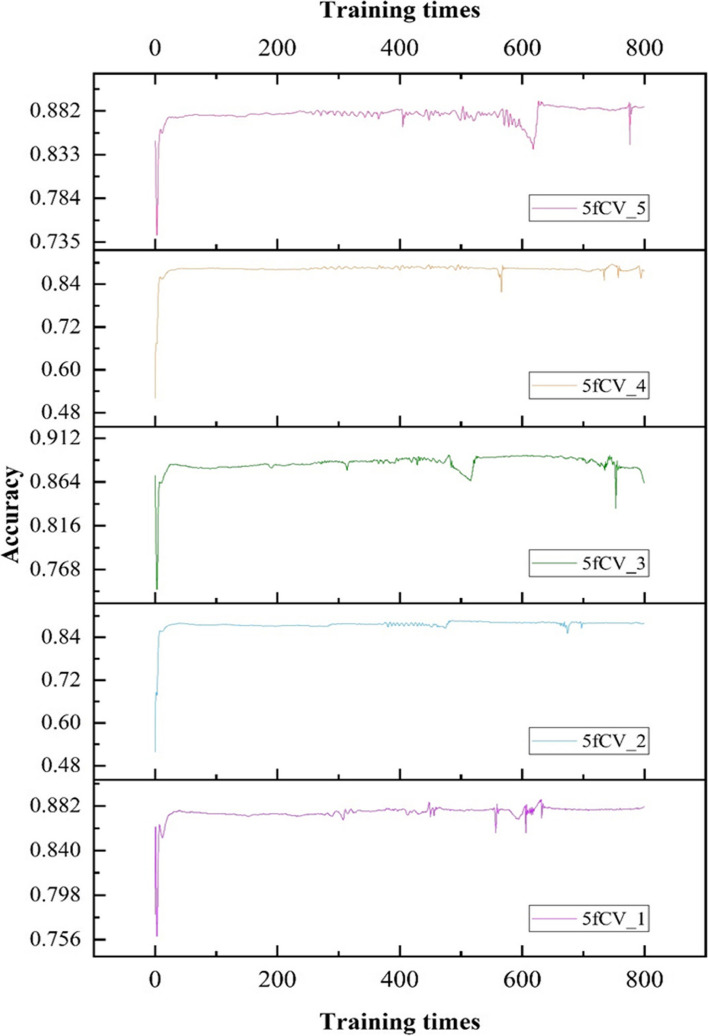
Accuracy statistics of model prediction results using miRNA-disease association matrix as input were obtained in the 5-fold cross-validation experiment, and 800 rounds of model training were performed in each fold experiment

**Fig. 9:**
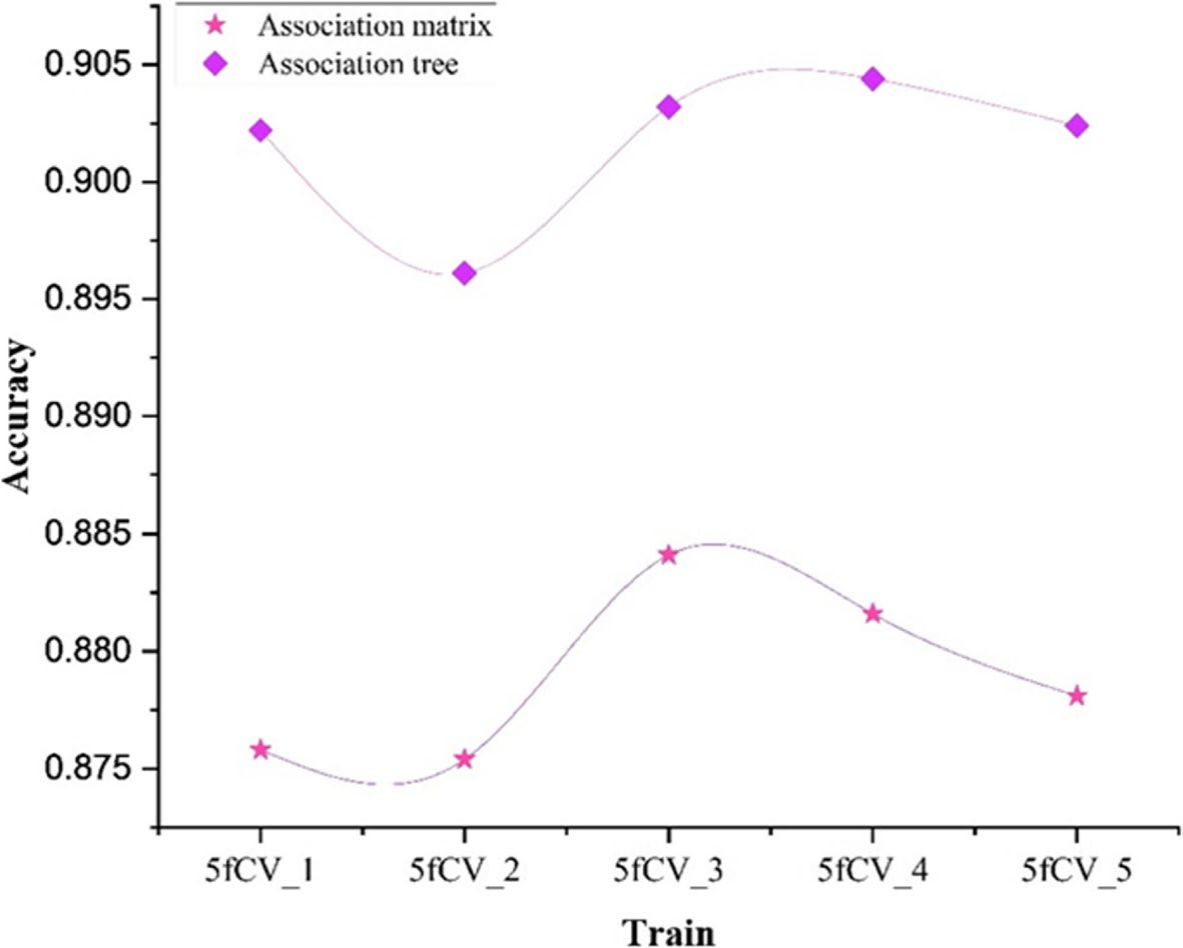
Comparison of accuracy results under 5-fold cross-validation experiment using association matrix and association tree as input

Comparing the model experimental results using accuracy as the evaluation parameter for models with association matrix and association tree as inputs, the results are shown in Figs. 8 and 9.

When training the model using the miRNA-disease association matrix as input, the accuracy remains below 0.9 in all cases, as shown in Fig. 8. The blue line in Fig. 9 represents the model trained with the association tree as input. In four out of five folds, the accuracy is better than 0.9, and all of them outperform the results obtained with the association matrix as input. This shows a significant improvement in accuracy. It can be concluded that by using the miRNA-disease association tree as input, a more reliable prediction model can be obtained, which can more accurately predict the potential miRNA-disease association.

### Analysis of experiments with the optimal model parameters

**Fig. 10:**
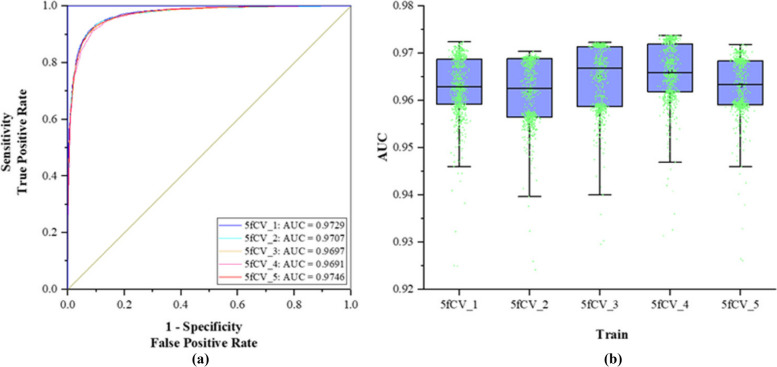
AUC experiment results of optimal parameters. **a** When the optimal parameters are used, the ROC curve under the experiment is 5-fold cross-verified, **b** Statistics of AUC results of TP-MDA model in 800 rounds of experiments

The TP-MDA model is trained with the optimal parameters under 5-fold cross-validation. ROC curves are plotted on the basis of the prediction results and the experimental results are statistically analyzed, as shown in Fig. 10.

The lowest AUC value in Fig. 10a reaches 0.9691 in the 5-fold cross-validation experiments. The statistical results in Fig. 10b show that more than 50% of the AUC values are greater than 0.97, indicating that this set of experimental parameters performs well during model training, leading to an improvement in the predictive performance of the model. At the same time, the model exhibits considerable stability across the entire dataset, avoiding the randomness of good model performance due to unbalanced sample selection. Compared to using the miRNA-disease association matrix as the model input, extracting the numerous node relationships from the association tree as feature vectors can result in a more accurate and superior prediction model for miRNA-disease associations.

**Fig. 11:**
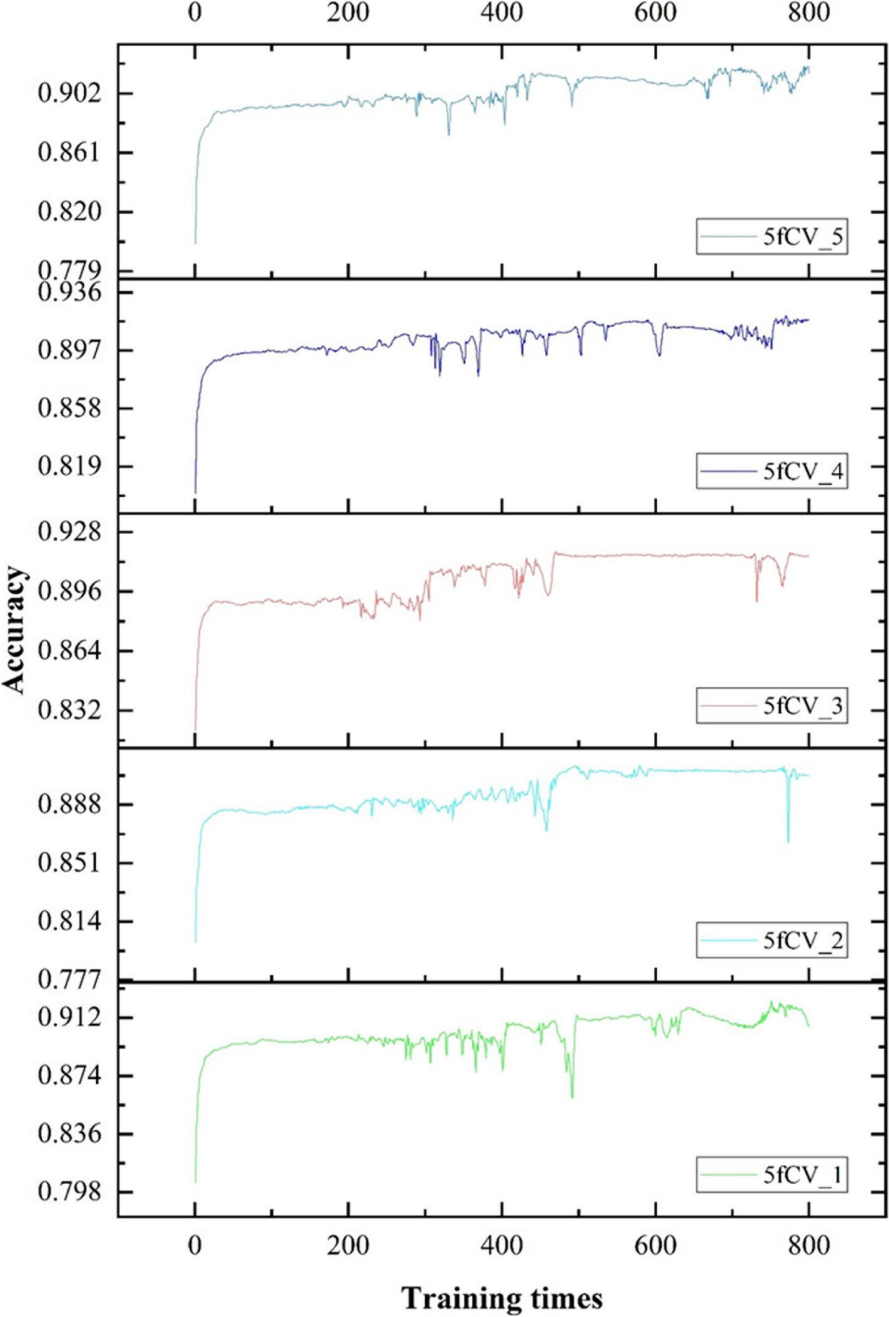
The accuracy statistics of the model were obtained by using the optimal parameters and the miRNA-disease association tree as input

**Fig. 12:**
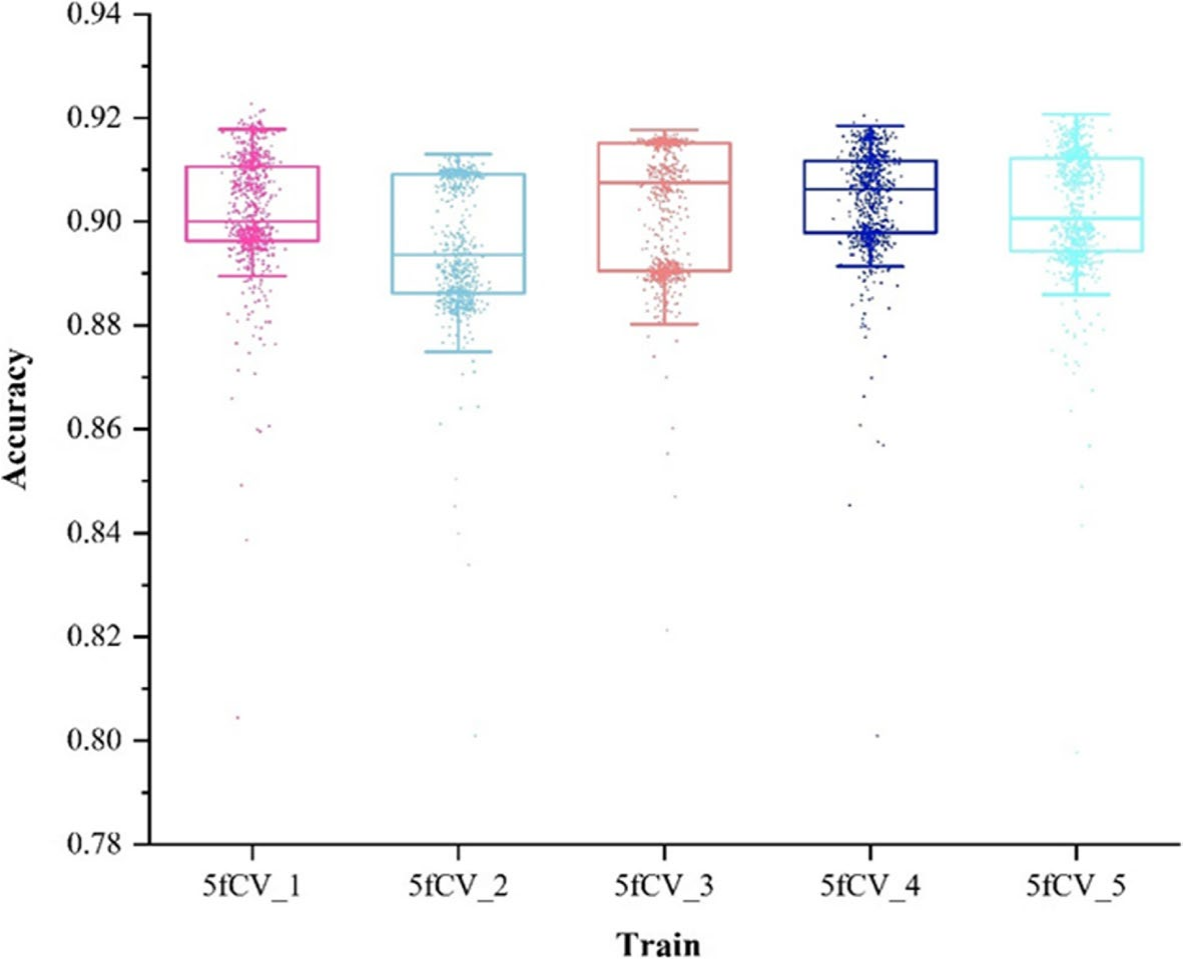
The accuracy distribution of the model was obtained by using the optimal parameters and the miRNA-disease association tree as input

Accuracy, as another parameter to evaluate, is critical to improving model performance. The changes in accuracy as the model is trained with optimal parameters are shown in Figs. 11 and 12.

There is a fluctuation in accuracy in each fold of the experiment, but the overall trend is upward and stabilizes around 600 training cycles, as shown in Fig. 11. The selection of these models for further training can have more reliable prediction results. The accuracy distribution of the 800 training cycles in a 5-fold cross-validation experiment is shown in Fig. 12. The highest accuracy is 0.9227. More than 50% of the data in the four folds exceed 0.9. The experimental results show that the model performs better when the parameters are optimized. This also confirms the stability and efficiency of the TP-MDA model.

### Comparison and analysis with other models

In this paper, TP-MDA was compared to three other miRNA-based models for predicting disease association using 5-fold cross validation. Comparison models are shown in Table 2.

**Table 2:** Introduction to comparative models

Modle	Details	Reference
MDPBMP	Through the construction of a heterogeneous miRNA-disease-gene information network, seven symmetric meta-paths are defined on the basis of different semantics. After constructing the initial feature vectors for all nodes, the vector information carried by nodes on meta path instances is extracted and aggregated to update the feature vectors of the initial nodes. After constructing the initial feature vectors for all nodes, the vector information carried by nodes on meta path instances is extracted and aggregated to update the initial node feature vectors. Finally, the miRNA and disease embedding feature vectors are used for the calculation of their respective relevance scores.	[23]
WBNPMD	The biased scores for miRNAs and diseases were constructed using the aggregated hierarchical clustering method. A bipartite network recommendation algorithm was then applied to assign transfer weights based on these biased scores to predict potential miRNA-disease associations.	[24]
BNPMDA	By combining known miRNA and disease similarities and properly configuring the initial information, transfer weights were constructed. Subsequently, potential miRNA-disease associations were inferred by means of a two-step bipartite network algorithm.	[25]

**Fig. 13:**
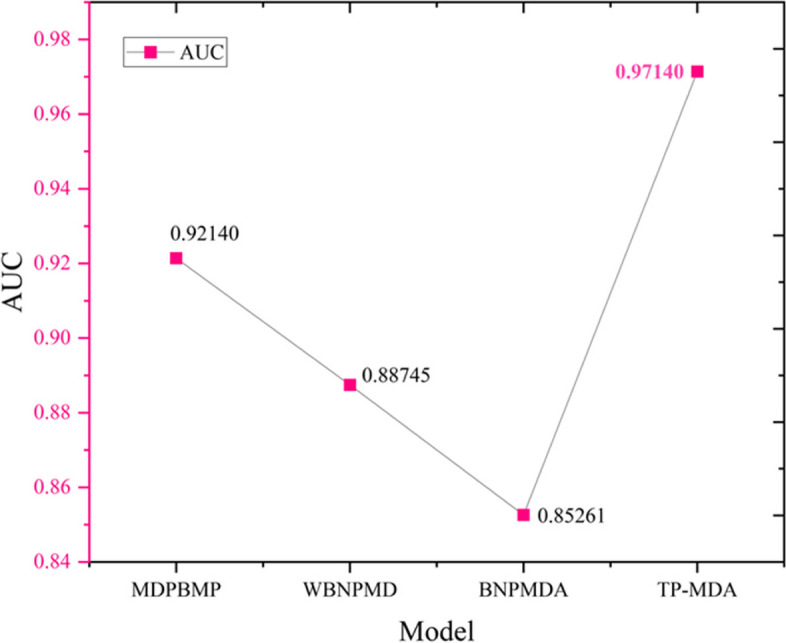
Graph comparing AUC values with other models

The comparison of the AUC results for the four different models is shown in Fig. 13.

TP-MDA obtained the highest AUC value. WBNPMD and BNPMDA had lower AUC values because they predicted miRNA-disease associations by resource allocation and transfer, which over-relied on the similarity matrix and affected their predictive performance. Compared to these two models, MDPBMP used 0.5 as the threshold to filter miRNA similarity, improved the reliability of similarity values, and increased the prediction accuracy by constructing feature vectors for nodes and aggregating information from all nodes in each meta path instance. The TP-MDA model presented in this paper does not rely on any known similarity measures. Instead, it uses the construction of a miRNA-disease association tree to describe the global relationships between nodes. It uses an efficient model to learn long dependencies within the association tree, resulting in a high-performing model with the highest AUC value.

## Case studies

For our case studies, we chose colorectal cancer [27–30] and lung cancer [31, 32], two common cancers. We used TP-MDA to score and rank the relevance of miRNA for unknown samples. The top 15 miRNAs were selected for validation by comparison with biomedical literature from the PubMed database. The predicted results of miRNA associated with colorectal cancer are shown in Table 3.

**Table 3:** The results of the association between colorectal cancer and miRNA

Rank	miRNA	PMID	Reference
1	hsa-mir-101-2	37,575,080	[33]
2	hsa-mir-29b-1	32,034,483	[34]
3	hsa-mir-181a-1	36,613,487	[35]
4	hsa-mir-769	30,565,566	[36]
5	hsa-mir-323a	31,238,337	[37]
6	hsa-mir-153-2	35,072,892	[38]
7	hsa-mir-193	33,317,596	[39]
8	hsa-mir-138-2	33,225,938	[40]
9	hsa-let-7f	36,295,073	[41]
10	hsa-mir-219	32,744,690	[42]
11	hsa-mir-663b	31,240,955	[43]
12	hsa-mir-1225	32,838,607	[44]

The validation results for lung cancer based on the predictions of the TP-MDA model are shown in Table 3. In the miRNA naming convention, “-1” and “-2” are added to the miRNA names to indicate that these miRNAs are transcribed and processed from DNA sequences on different chromosomes but share the same mature sequence [45]. Therefore, even though the top-ranked miRNA, hsa-mir-101-2, hasn’t been directly validated to be associated with colorectal cancer, it is known that miRNA hsa-mir-101, which shares the same mature sequence, is associated with colorectal cancer. Therefore, there is an association between the miRNA hsa-mir-101-2 and colorectal cancer. In summary, of the top 15 miRNAs predicted to be associated with colorectal cancer by TP-MDA, 12 were validated.

The prediction results of miRNA associated with lung cancer are shown in Table 4:

**Table 4:** Results of the association between lung cancer and miRNA

Rank	miRNA	PMID	Reference
1	hsa-mir-181a	32,506,887	[46]
2	hsa-mir-22	32,514,270	[47]
3	hsa-mir-25	35,628,157	[48]
4	hsa-mir-130b	31,389,608	[49]
5	hsa-mir-30b	37,686,123	[50]
6	hsa-mir-27a	31,772,627	[51]
7	hsa-mir-342	32,938,459	[52]
8	hsa-mir-708	31,419,576	[53]
9	hsa-mir-218	35,034,634	[54]
10	hsa-mir-27b	31,772,627	[51]
11	hsa-let-7c	36,388,933	[55]
12	hsa-mir-128	34,533,066	[56]
13	hsa-mir-125b-2	31,959,728	[57]
14	hsa-mir-219	32,159,887	[58]
15	hsa-mir-486	30,963,622	[59]

**Fig. 14:**
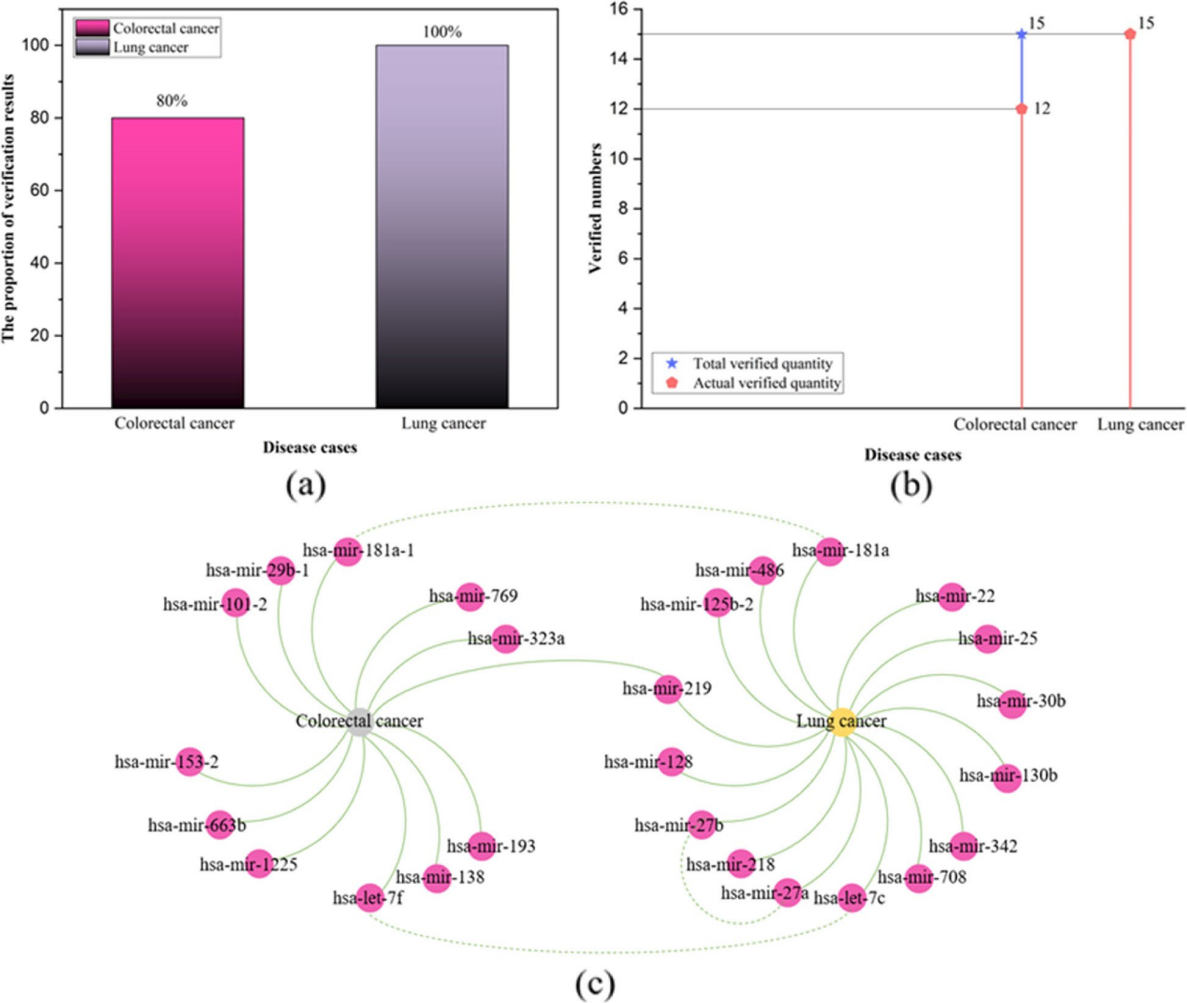
Case study results statistics and visualization. **a** The proportion of the number of results obtained by actual verification, **b** Number of validation results, **c** Validation result visualization

The top 15 miRNAs predicted to be associated with lung cancer by the TP-MDA model are shown in Table 4. Among them, the sixth ranked miRNA, hsa-mir-30b, and the tenth ranked miRNA, hsa-mir-30b, share a high degree of sequence homology. The eleventh ranked miRNA, hsa-let-7c, follows an earlier nomenclature and is primarily used to represent the let-7 miRNA family. The study by Yin et al. [60] demonstrated that the let-7 miRNA family is involved in the regulation of resistance to epidermal growth factor receptor tyrosine kinase inhibitors (EGFR-TKIs) and may serve as predictive biomarker for EGFR-TKI resistance in non-small cell lung cancer (NSCLC). EGFR-TKI resistance represents a significant challenge in treating NSCLC. In summary, all of the top 15 miRNAs predicted to be associated with lung cancer by TP-MDA were validated. The statistics and visualization of the verification results are shown in Fig. 14.

Among the top 15 miRNAs associated with colorectal cancer and lung cancer, 12 and 15 miRNAs were validated, accounting for 80% and 100% of the total validated miRNAs, respectively, as shown in Fig. 12a and b. Among the top 15 predicted miRNAs associated with colorectal cancer and lung cancer, hsa-mir-219 is associated with both diseases simultaneously, as shown in Fig. 14c. The miRNA hsa-mir-181a-1, which is associated with colorectal cancer, shares the same mature sequence with hsa-mir-181a, which is associated with lung cancer. In addition, the hsa-let-7 family members, hsa-let-7f and hsa-let-7c, are associated with colorectal cancer and lung cancer, respectively. This suggests that the relationships between miRNAs and diseases are complex and that the TP-MDA model has the ability to predict complex associations between miRNAs and diseases.

## Conclusions

This paper introduces the TP-MDA miRNA-disease association prediction model. This model does not rely on any similarity measures and employs a multi-head self-attention mechanism to extract global vector information from the miRNA-disease association tree. Finally, the model is trained using a FANN framework in a 5-fold cross-validation experiment. The experimental results show that this algorithm performs excellently in predicting miRNA-disease associations. It shows good and stable performance in cross-validation. Compared with other models, it has better prediction effect. The TP-MDA model can serve as a reference method for data mining and association prediction in various fields, including life sciences, biology, and medical genetics. However, the field of miRNA-disease association prediction still needs to be further explored despite the positive experimental results. For example, understanding the complex interactions between different biological information in disease mechanisms is a significant challenge. In future work, the development of algorithms capable of handling multiple types of biological information will be critical to achieving more accurate and effective predictions in this area.

## Data Availability

The codes, architecture, parameters, dataset, functions, usage and output of the proposed model are available free of charge at GitHub. (https://github.com/BiyuHou/miRNA-disease.git).
